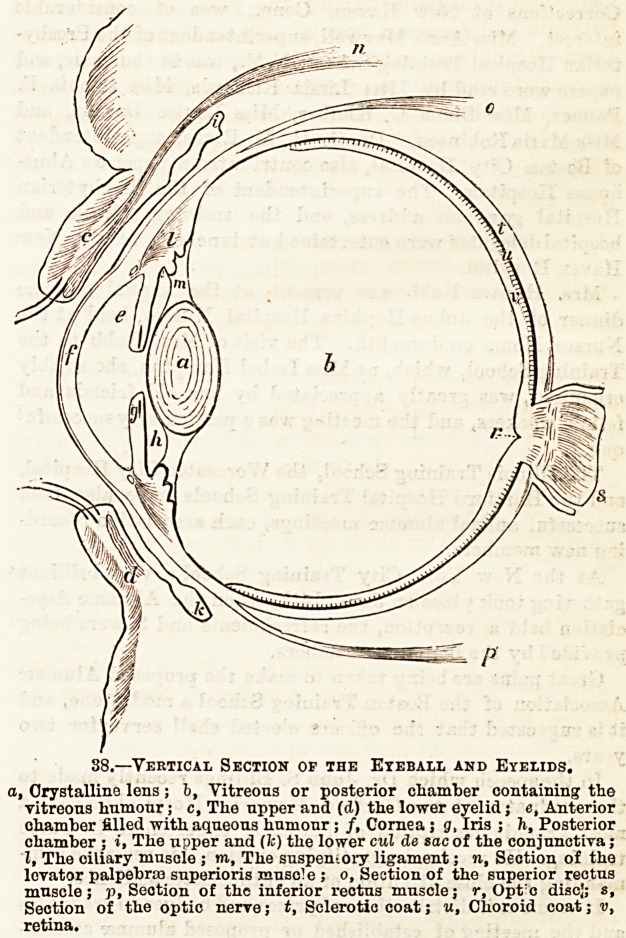# The Hospital Nursing Supplement

**Published:** 1895-07-20

**Authors:** 


### "The Hospital" Nursing Mirror.

Being the Extra Nursing Supplement of "The Hospital" Newspaper.

[Contributions for this Supplement should be addressed to the Editor, The Hospital, 428, Strand, London, W.C., and should have the word "Nursing" plainly written in left-hand top corner of the envelope.]

### News from the Nursing World.

### IN THE SERVICE OF THE QUEEN.

Her Majesty the Queen has all her life shown affectionate regard for those who have the honour of being employed in her household, and evidence is not wanting that Her Majesty regards the death of a faithful subject as a serious loss to herself. Early last week the Queen's Highland attendant, Mr. Francis Clarke, who suffered from cancer in the throat, died at Buckingham Palace. Everything surgically possible was done for the sick man, and two experienced nurses were engaged through the Nurses' Co-operation, 8, New Cavendish Street. A few days after Mr. Clarke's death both nurses were summoned to Windsor Castle where they had the honour of an interview with Her Majesty, who received them most kindly, and later on they were entertained at tea and taken through many of the beautiful and interesting rooms before leaving the Castle. Nurse Hine and Nurse Theobald both became members of the Nurses' Co-operation during its first year of existence. Nurse Hine was trained at the North-Eastern Children's Hospital and at St. Bartholomew's, and Nurse Theobald received her training at the Children's Hospital, Shadwell, and at the London Hospital. Both have had considerable experience in private nursing.

### OUR PRINCESS.

"Is the day fixed?" has been eagerly asked by many a nurse to whom the anticipated visit to Marlborough House must ever rank as an event of unparalleled interest. The question has been affirmatively answered by an announcement that her Royal Highness the Princess of Wales has named Friday, July 26th, for her reception at Marlborough House of the Third and Fourth Thousand nurses belonging to the Royal National Pension Fund. Each will receive her certificate from the hands of "Our Princess," as the First and Second Thousand policyholders were in their turn privileged to do. The pleasure and lifelong gratitude expressed by the nurses who flock from all parts of the realm to this reception can hardly be realised in their entirety by their Royal hostess. The gracious and kindly hospitality exercised at Marlborough House forms another link in that chain of loving loyalty which binds the hearts of her own special nurses to their Royal President the Princess of Wales.

### AT THE QUEEN'S HALL.

A preliminary meeting of the Third and Fourth Thousand of the Royal National Pension Fund nurses will be held on Friday, July 26th, at half-past ten a.m., in the large Queen's Hall, Langham Place. The beautiful badges in the Danish colours which have been prepared, with the approval of the Princess of Wales, will be worn by Her Royal Highness's nurses for the first time on this occasion. It is hoped that nurses will allow themselves plenty of time for reaching the hall (which is so conveniently situated near to Oxford Circus), as trains and omnibuses are liable to be crowded, hence to secure punctuality in arriving, a margin should be allowed for unavoidable delays. The early hour fixed for the preliminary meeting ensures sufficient time after it for nurses to get rest and refreshments before proceeding to the reception at Marlborough House. Indoor uniform will be worn, except in the case of District Nurses, who can appear in bonnets if they prefer to do so, but without cloaks.

### ATTENTION.

It is particularly requested that all nurses strictly adhere to the directions given to them, as this alone can ensure the successful carrying out of arrangements which have been most carefully planned for their convenience. Light refreshments will be provided at the hall, and a service of omnibuses will probably be secured to convey the nurses from thence to Marlborough House. The railway companies have generously consented to issue return tickets at single fares, and no pains have been spared to make the day in every way a complete success.

### ASYLUM ATTENDANTS AND NURSES.

It is announced that at the inaugural meeting of the Association of Asylum Workers, which takes place this week, Sir Benjamin Ward Richardson will be in the chair. Dr. Walmsley has been appointed honorary secretary to the new society, which aspires to raise the status of attendants, who, according to the last census, number 6,000 males and 15,000 females employed in asylums. The names of those in favour of the association include the Earl and Countess of Arran, Sir William Broadbent, many medical superintendents of asylums, and other doctors, also a number of head nurses. The objects of the association are excellent, and deserve the hearty co-operation of all interested in securing skilled attendants for the insane.

### SHOULD CHILDREN BE ADMITTED?

The throwing open of all departments of hospitals for inspection on fête days is in the abstract highly to be commended, for it is seldom found that closed doors betoken successful internal administration. Yet care must be taken that the privilege of inspection be not abused by those to whom it is granted, and there seems a tendency for curiosity to outrun discretion when children are taken by their friends into hospital museums. In one of the best-known anatomical collections in the world, on a recent fête day, two or three children and various very young girls were to be observed gazing with open-eyed wonder at "those dreadful things" in glass cases. These models are admirably realistic studies for students, but they form an equally sure foundation for the future dreams of sensitive and juvenile spectators. The taste of adults who take children into such exhibitions is too bad to be styled "questionable."

### DISTRICT NURSES AT PLAISTOW.

The second annual meeting of the Plaistow Maternity Charity and District Nurses' Home was held at Lord Brassey's house in Park Lane, and was exceedingly well attended. The report showed that 63,000 visits had been paid during the year, and that 2,081 patients had received special day and night nursing. Lord Winchilsea, who was in the chair, pleaded for increased financial support for a charity of which the work covered so large and poor an area. The annual subscriptions need to be largely augmented, a sum of £800 being also urgently required to pay off a mortgage on the Home.

### THE USE OF A SHEET.

"But where's the clean sheet gone?" asked the District Nurse. "I left you looking so nice and tidy, Mrs. Brown, and now I declare you have quite unsettled your bed and the sheet's disappeared altogether!" "Oh, don't you put yourself about, nurse," said the sick woman in a conciliatory tone, "your sheet as you lends me is folded up atop o' they chest of drawers. You've come a bit early to-night, or you'd a-found 'im on all right." Nurse gazed at the woman inquiringly; "I don't understand you," she remarked. The sick woman smiled, "Well, nurse," she said, "I knows you likes to see me a lookin' clean and wholesome, so I just puts that sheet over me when its gettin' to your time for coming. I 'as 'im took off as soon as you're gone, for I ain't so wasteful as to use a clean sheet when there ain't nobody to see it! I just keeps the old 'un 'andy and uses 'im."

### A MAID OF ALL WORK.

When alterations in the nursing department are contemplated at an institution it is certainly wise for the committee to acquaint themselves with the existing arrangements of establishments similar to their own. Comparisons of facts and figures are helpful, and the relative positions held by the nurses in different places may be taken in the light of example or warning. We have often condemned the mixed duties allotted to nurses in country unions, but even to us it is a novelty to find that the second nurse at Llanfyllin Union has to "attend infants, and act as general servant." This transpired (as reported by the local press) from information collected by the Clerk to the Newtown and Llanidloes Board of Guardians regarding the proportion of inmates to nurses in rural unions.

### DISTRICT NURSING AT BROUGHTY FERRY.

The financial support given to the Broughty Ferry Nursing Fund last year was not quite so good as in the previous twelve months. The record of work for the last six months shows that 1,162 visits were paid during that period by Nurse Watson to 43 sick poor in their own homes.

### ATTENDANTS' WAGES IN SLIGO.

In the local report of a meeting held recently at Boyle, the chairman is said to have remarked that the lunatic female nurse received £6 a year at Sligo, and a "lunatic keeper on the male side" £5. It seems improbable that a woman content with such wages can be a nurse in anything but name, and surely she cannot be expected to have had any special training for her responsible work. The term "keeper" appears strangely out of place in connection with humanity, being ordinarily associated with persons in charge of wild animals. For menagerie work intelligence and knowledge are requisite, and probably command far better pay than that received by the attendant placed in charge of sufferers from brain disease at Sligo.

### AT THE CRYSTAL PALACE.

Medallions and certificates were last week presented by Her Royal Highness Princess Christian to members of the Norwood Centre of the St. John Ambulance Association. Princess Christian was accompanied by her daughter, Princess Victoria, and the ceremony, which took place at the Crystal Palace, was an interesting one.

### MATRONS AND DOCTORS.

The recent meeting held between a dozen medical men and the same number of matrons, members of the R.B.N.A., does not appear to have had any particular influence on the present position of the association. It seems to have been convened quite unofficially at a private house for the purpose of discussing the bye-laws, which, as now worded, require that *ex-officio* as well as other nurse-members shall take their turn in retiring annually. Members are eligible for re-election at the end of twelve months, but in the meantime, amongst those who have to undergo this compulsory temporary retirement from the council, Miss Thorold, Miss Stewart, Miss Hagg, Miss Loch, and Mrs. Bedford Fenwick are mentioned. It is said that all questions relating to the alteration of the bye-laws will be postponed until the autumn.

### REGISTERED NURSES' SOCIETY.

The report of the Registered Nurses' Society, lately prepared, is not yet ready, we learn, for general distribution. Miss de Pledge and Mr. Brudenell Carter have retired from the committee. Conditions of membership include the payment of an entrance fee of a guinea on the part of all selected candidates. In this and in charging three guineas a week for infectious cases, the society differs from the Nurses' Co-operation, 8, New Cavendish Street; but some of its rules appear to be framed on the model of the older association, which, by its success, has naturally encouraged imitation.

### A SATISFACTORY COLLECTION.

Eloquent testimony to the value of the work done by the Taunton District Nursing Association was given by the Rev. S. H. King in the course of his sermon on the occasion of the Friendly Societies' Church Parade. The preacher commended the noble and Christian work which supplemented, but could not in any way supplant, that done by the hospital, doctors and clergy in Taunton. The arrangements for the parade were admirable, and they resulted in an addition of about £40 to the funds of the association.

### SHORT ITEMS.

An order has been issued throughout the Punjab army that all drinking water used in barracks and Government hospitals must be previously boiled. During the few months that this has been done at Mian Mir, one of the most unhealthy stations in India, there have been no serious cases of fever notified.—The Guardians at Kenmare have agreed, on the advice of their Medical Officer, to engage two permanent nurses, one for day the other for night duty.—The Amalgamated Friendly Societies of Worthing have postponed Hospital Saturday till the 31st of August.

### Elementary Anatomy and Surgery for Nurses.

By W. McAdam Eccles, M.B., M.S., F.R.C.S., Lecturer to Nurses, West London Hospital, &c.

### THE SPECIAL SENSES.

The special senses are five in number, viz.: Sight, Hearing, Taste, Smell, and Touch. Each of these, with the possible exception of touch, will be found to have a special sense organ associated with it, and in this, the last chapter on elementary anatomy proper, these several organs will be briefly described.

The sense of *Sight* has for its organ the eye, an example of most beautiful mechanism. The eyeball, with its muscles, vessels, and nerves is situated in the orbit surrounded by a good deal of fat, which serves to fill up all the spaces and thus to protect the eye. In front of the orbit are the two eyelids; each has a firm cartilage in it, with cilia or eyelashes along the margin of the lid. The outer surface presents skin, while the inner is lined by a smooth membrane, the conjunctiva, which is also reflected over the front of the eyeball. The lids are closed by a circular muscle called the orbicularis, and the upper lid elevated by a special muscle. The surface of the conjunctiva is kept moist by the secretion of a gland, the lachrymal or tear gland, placed at the upper and outer part of the orbit. This fluid, known as tears, passing over the front of the eye is collected by two minute holes, one in each lid near the inner side. Each of these puncta lead into small canals which themselves empty into the lachrymal sac which communicates with the nose by the lachrymal or nasal duct. (See Fig. 37.) When the secretion of tears is excessive, as in crying, all the secretion is unable to be carried away by the apparatus just described; consequently tears flow over the cheek. The eyeball, somewhat globular in shape, measures about one inch in diameter. The front part is formed by the anterior chamber, and is smaller, and projects in front of the posterior much larger portion composed of the posterior chamber. Between the two lies the lens with its capsule. (See Fig. 38.) The eyeball has three coats. The external is the sclerotic, which is continuous in front with the cornea; the middle is called the choroid, and contains the bloodvessels; while the internal is the retina. The sclerotic is a thick, very tough fibrous coat, and forms the white of the eye. The cornea is the beautifully transparent part of the eye in front, within which is the anterior chamber containing the aqueous humour and the coloured iris or curtain in front of the lens. (See Fig. 38) The opening in the centre of the iris, which appears as a dark circular aperture in the

Fig. 37.—The Lachrymal Apparatus.

normal eye, is known as the pupil, and varies in size owing chiefly to its contraction or relaxation according to the amount of light shining through it. The crystalline lens is another transparent medium lying behind the iris and surrounded by a capsule, which can be tightened or relaxed by a small muscle called the ciliary muscle, and thus the curvature of the lens can be altered. (See Fig. 38.) Behind the lens is the posterior chamber which is shut off from the anterior, and contains a clear viscid substance known as the vitreous humour. The optic nerve reaches the eyeball behind and somewhat to the inner side, and the fibres piercing the outer

38.—Vertical Section of the Eyeball and Eyelids.

*a*, Crystalline lens; *b*, Vitreous or posterior chamber containing the vitreous humour; *c*, The upper and (*d*) the lower eyelid; *e*, Anterior chamber filled with aqueous humour; *f*, Cornea; *g*, Iris; *h*, Posterior chamber; *i*, The upper and (*k*) the lower *cul de sac* of the conjunctiva; *l*, The ciliary muscle; *m*, The suspensory ligament; *n*, Section of the levator palpebræ superioris muscle; *o*, Section of the superior rectus muscle; *p*, Section of the inferior rectus muscle; *r*, Optic disc; *s*, Section of the optic nerve; *t*, Sclerotic coat; *u*, Choroid coat; *v*, retina.

and middle coats terminate in the retina. The eyeball is acted upon by no less than six muscles, which can turn it in all directions.

### Appointments.

The Hospital, Tewkesbury.—Miss Gibbons has been appointed Matron of this hospital. She received her training at the Middlesex Hospital, and was afterwards assistant nurse at Tewkesbury Hospital. Miss Gibbons is therefore well known to those amongst whom her future work lies, and we congratulate her on her appointment.

Falmouth Hospital and Dispensary.—Miss Elizabeth Nichol has been appointed matron of this hospital. She was trained at St. Mary's Hospital for Women and Children, Manchester, and at Oldham Infirmary. Miss Nichol then did private nursing in connection with the Oldham Association, and was charge nurse at Sheffield Union Infirmary. We wish her every success in her new work.

### Presentation.

The occasion of Miss Baxter's retirement from the post of lady superintendent at the Royal Berks Hospital was pleasantly commemorated by a tea-party at which were present many members of the staff and other friends. The chaplain presented to Miss Baxter, in the name of all connected with the hospital, a silver tray, epergne, egg holder, &c., and an address which proved the affectionate esteem with which she is universally regarded. For twenty-four years Miss Baxter has fulfilled her numerous duties in a way which has won for her many friends.

### Our American Letter.

(Contributed.)

The nursing section, which occupied the second of the two days devoted to the National Conference of Charities and Corrections at New Haven, Conn., was of considerable interest. Miss Anna Maxwell, superintendent of the Presbyterian Hospital Training School, N.Y., was in the chair, and papers were read by Miss Linda Richards, Miss Sophia F. Palmer, Miss Diana C. Kimber, Miss Louise Darche, and Miss Maria Robinson. Dr. G. H. M. Rowe, superintendent of Boston City Hospital, also contributed a paper on Almshouse Hospitals. The superintendent of the Presbyterian Hospital gave an address, and the training schools and hospital delegates were entertained at luncheon at the New Haven Hospital.

Mrs. Hunter Robb was present at the annual alumnæ dinner of the Johns Hopkins Hospital Nurses, held at the Nurses' Home on June 6th. The visit of Mrs. Robb to the Training School, which, as Miss Isabel Hampton, she so ably organised, was greatly appreciated by her old friends and fellow workers, and the meeting was a particularly successful one.

The Illinois Training School, the Worcester City Hospital, and the Hartford Hospital Training Schools have also held successful annual alumnæ meetings, each association recording new members.

At the New York City Training School a very brilliant gathering took place on June 14th, when the Alumnæ Association held a reception, the refreshments and flowers being provided by the honorary members.

Great pains are being taken to make the proposed Alumnæ Association of the Boston Training School a model one, and it is suggested that the officers elected shall serve for two years.

In the speech which Dr. John S. Billings recently made to the graduates of the Garfield Memorial Hospital he gave a most graphic description of his early experiences in nurse training. He received a gratifying ovation, his remarks commanding the close attention of an enthusiastic audience.

It seems as if distributions of prizes and badges to graduates and the meeting of established or proposed alumnæ associations were our only items of news just now; but the scheme for a pension fund for nurses is also attracting a considerable amount of attention over here. The *Trained Nurse* has opened its columns to correspondence on the subject, and so far the majority of writers appear to favour the plan of each school providing by means of its own alumnæ association for its sick and disabled nurses. The project started recently at Philadelphia must receive full consideration before a decision can be arrived at as to the comparative value of national or individual beneficence. A loss of independence appears to be dreaded by the majority of the nurses in America, who imagine that "a national movement" must necessarily be a pauperising one. The number of trained nurses in the United States being comparatively small it is not difficult at present to provide assistance for invalids. The graduates who pass from the training schools in annually increasing numbers may in the future find sickness and old age less easily provided for.

### Wants and Workers.

[The attention of correspondents is directed to the fact that "Helps in Sickness and to Health" (Scientific Press 428, Strand) will enable them promptly to find the most suitable accommodation for difficult or special cases.]

The District Nurse, Marazion, Cornwall, would be glad of gifts of old linen for dressings and poultices.

Can anyone inform me if there is a home where an elderly woman, who is blind, would be received for eight or nine shillings a week?—Sister Emily.

### New South Wales.

### AN AUSTRALIAN POORHOUSE.

By a Former Nurse.

Newington Asylum has been aptly described as "a Paradise for old women." It is built on rising ground facing the Paramatta River on the former site of a convict settlement. The asylum consists of a vast range of buildings, and the inmates are classed according to their infirmities or otherwise. There are Protestant and Catholic hospitals, each containing thirty-six beds, convalescent wards, under the immediate control of the nurses, and other dormitories. Those on the ground floor are for cripples and the blind; those upstairs for the more able-bodied, in charge of wardswomen. Six trained nurses, each receiving from £45 to £50 a year with uniform and house accommodation, divide the care of the sick between them. Two day nurses and two night nurses are in each hospital, the night nurses being in charge of the whole asylum. Two nurses, in addition to the convalescent ward, have charge of cancer and "sore legs" wards, also they superintend the "Loonies" yard. This is a truly Australian term for a high fenced-in grass square, with an octagonal summer house in the centre, fitted with stove, table, seats, and a cupboard containing dishes for meals. Here all harmless lunatics and imbeciles are kept in the daytime, to prevent their annoying or being hurt by stronger sensible inmates. Two women helpers, as they are called, take charge under the nurses' supervision. At night the people go back to their several dormitories.

The laundry is worked by able-bodied women who get threepence a day, and is most perfectly arranged with hot and cold taps over each trough or sink for washing. It is supplied with a hot-air press for drying on wet days, consequently there is an endless supply of clean clothes, and the hospitals look very nice with spotless white counterpanes and bed linen, the Government red stripe running through all. The public bath-room contains twelve cemented baths with hot and cold water, the brass taps polished till they shine again, the floor being as white as snow. The dining-hall, gaily decorated with hanging lanterns and fans, capable of seating over 200, contains several tables each in charge of a mess-woman who is responsible for the order and neatness of her table and the comfort of the diners. The lady superintendent, Mrs. Murray, formerly matron of the Prince Alfred Hospital, Sydney, is an educated gentlewoman, and seems in every sense the right woman in the right place. This wonderful place has developed greatly under her management. Before her time it was governed more on the principle of a convict settlement, beer, tobacco, and brandy being the rewards for industry, whilst bad language and fighting were general. Now no stimulant is given without the doctor's orders, and the diet, which was formerly of the coarsest description, is well suited to the requirements of each individual, milk, butter, eggs, fruit, and vegetables being supplied daily. A sub-matron is responsible for the food and its cooking, and another sub-matron for the inmates' reception, their clothing and bathing, and also for reporting any cases of sickness in the dormitories. A dispenser lives on the premises; a doctor is in attendance daily. The staff has been recently increased to eight nurses, who have comfortable quarters, good food, a boat for recreation, a garden of their own, and the use of a horse and buggy.

### Where to Go.

Bazaar in the Grounds of Dunrobin Castle on September 5th and 6th, in aid of the Sutherland District Nursing Association.

### Everybody's Opinion.

[Correspondence on all subjects is invited, but we cannot in any way be responsible for the opinions expressed by our correspondents. No communications can be entertained if the name and address of the correspondent is not given, or unless one side of the paper only be written on.]

### "NURSING ASSOCIATIONS."

LORD KINNAIRD writes, from 2, Adelphi Terrace, Strand, London: In your issue of the 13th appears a letter from Miss Amy Hughes, late superintendent of the Central Training Home, Bloomsbury, which contains by implication so many and such serious mis-statements regarding the training, work, and methods of the nurses attached to the Nursing Association, having its offices at 2, Adelphi Terrace, Strand, and of which Mrs. Selfe Leonard is the honorary superintendent, that I must ask you to allow me a short reply. Considering the vast area to be covered, it is to be regretted that a lady connected with the Bloomsbury nurses is not content that they should do their own good work without detracting from and sometimes seeking to supplant the equally good work of others. I may add that our association is that named in the bequest of the late Mr. C. Soames (not as misprinted, Mr. Durham), which is referred to by Miss Hughes; it and its methods have been adopted by him for the nursing of the Greenwich parishes, after trial of the working of both the Bloomsbury Association and our own. Miss Hughes, in her letter, states by clear implication that our association is sectarian, that our nurses are not as well trained in hospital work as are the Queen's nurses; and do not similarly work under trained superintendence and inspection. These statements are incorrect. As regards the implied charges of sectarianism, all our nurses are unsectarian in the usual sense of that word; that is to say, the association employs nurses of all Christian denominations, who attend the sick poor absolutely without distinction of religion or creed. While it is true that years ago practically untrained nurses were employed by the above mission, yet this has long been ancient history. It must be remembered that this association took its rise before the development of modern nursing, our association and the East London Nursing Association being the pioneers of district nursing. Many of our nurses have received three and four years' hospital training, while none are accepted who have not had at least one year's training in a general hospital, to which is added a monthly nurse training in a lying-in hospital and subsequent training in district nursing. According to the last report, the Queen's Nurses receive "six months' training and experience in district nursing, in addition to a minimum of twelve months' training, which they have already received in a general hospital." Our nurses are all under the supervision and inspection of highly-trained ladies, who have many of them worked for years in such hospitals as St. Thomas's, St. Bartholomew's, and Guy's. Each of these superintending sisters has the charge of a small group of nurses, and corresponds in district nursing to the sister in the hospital ward. This has also long been the feature of our mission, as we have believed with Miss Hughes that "it is essential to keep the nursing at a uniform standard and to prevent the almost inevitable deterioration which comes when nurses, however good, are left without trained supervision." Our nurses work in their 78 districts under the local doctors, with whom they are immediately put into communication. Forms to receive the doctors' written orders are left at every case. It was under the auspices of this mission that the nursing of the extern maternity work for St. Bartholomew's Hospital commenced. Miss Hughes heard Mrs. Leonard's account of this special form of her nursing at a conference in South London, and in a few months the mission received an intimation from the doctors of St. Bartholomew's that "the Metropolitans had volunteered to do this work for nothing," Mrs. Leonard's nurses having been generously supported by two members of the St. Bartholomew's staff after a period in which they also had been voluntarily supplied. The extern maternity cases of St. Thomas's and other hospitals are still nursed by the nurses of our association, to the absolute satisfaction of the staff and governing bodies. The original idea of district nursing for the extern maternity cases attended from hospitals originated in our association some sixteen years ago. We regret the necessity for this letter. It has always been our wish to work amicably with the other associations, who are doing so much good among the poor. We have no desire to enter into any rivalry with other nurses, except in the mutual endeavour to promote good nursing and the welfare of the sick.

### IS CYCLING AN AMUSEMENT FOR NURSES?

"AN OLD NURSE WHO IS INTERESTED IN YOUNG ONES," writes: Perhaps I am somewhat behind the times, for I have a decided objection to the "new woman," and to many of the amusements into which she has launched out. Cycling I have looked on as unfit for any woman, and at the thought of nurses cycling have held up my hands in horror. Now for the last month I have been enjoying a much-needed holiday in a country village not far from Manchester, and walking along a country road one day I saw a lady cyclist coming towards me. Thought I, I will have a good look at this strong-minded female. As she approached and passed on, to my amazement I recognised in this "new woman" one of the most capable nurses with whom I have ever come in contact, and, moreover, a most sensible and clear-headed woman. The last time I had seen her was by the bedside of a dear friend of my own who was dying in a far northern county hospital. The cool courage she then displayed under most trying circumstances made me thank God that there were such women to tend the sick and dying. Now I saw this same woman riding a bicycle! It was, indeed, a shock to me, and I could think of nothing else for days. Shall I be thought very fickle when I own that after seeing that one nurse on a cycle, plainly dressed in a tweed costume with a skirt, my ideas of the new woman have been entirely revolutionised? To nurses I would cordially say, cycle by all means when you can, for it seems a good healthy kind of exercise.

### Notes and Queries.

### Queries.

(205) *Ward Linen.*—Will you tell me the quantity of linen, &c., required for a surgical and accident ward?—*Nurse.*

(206) *Dictionary.*—Would the correct pronunciation of medical terms be facilitated by the study of "Hoblyn's Dictionary," which costs, I believe, about half a guinea, less discount?—*E. M.*

(207) *Inebriates.*—Can you give me addresses of some homes for inebriates?—*A B C.*

(208) *Medical.*—I shall be very grateful if you will furnish me, through your answers to queries, with the addresses of several medical electricians of repute.—*Nurse A.*

(209) *District.*—Where can I get the names of district nursing homes in London?—*A. J. N. W.*

### Answers.

(205) *Ward Linen (Nurse).*—Sheets, 5; blankets, 3; counterpane, 1 (two over in each ward); pillow cases, 3; draw sheets, 4 (children's ward, 6); doctor's towels, 12 per ward; round towels, 6 per ward; table cloths, 4 per ward; tea cloths, 6 per ward. (From Burdett's "Cottage Hospitals.")

(206) *Dictionary (E. M.).*—It would certainly help you. A second-hand copy can sometimes be procured.

(207) *Inebriates (A B C).*—You will find the addresses you want on p. 828 in "Burdett's Hospital Annual" for 1895.

(208) *Medical (Nurse A.).*—We advise your consulting your own medical man, if you require treatment yourself. If you desire to get instruction in electricity and massage, you had better apply to the Hon. Secretary of the Society of Trained Masseuses at 12, Buckingham Street, Strand, enclosing a stamped envelope for reply.

(209) *District (A. J. N. W.).*—In "Burdett's Hospital Annual," p. 617. You can get information of the Queen's Jubilee Nurses' Home from Miss Peters, St. Katharine's Hospital, Regent's Park.

### Royal British Nurses' Association.

Arrangements for Wednesday, July 24.

The Secretary of the Royal British Nurses' Association requests us to state that it is hoped all members attending the reception to be held by H.R.H. the President, at the Queen's Hall, Langham Place, will bring their cards of invitation with them, as it is Her Royal Highness's graciously expressed wish that each member shall be personally presented on arrival, and the presentation of the cards of invitation on entering the hall will greatly facilitate this ceremony, bearing as they do the name and title of the member to whom each was addressed. Outdoor uniform or ordinary dress will be worn.

Lending and Reference Library.

Arrangements in connection with the formation of a Lending and Reference Library are progressing. The book cases are on order, estimates having been submitted to, selected and approved by the Executive Committee; rules are being printed, and will be published in the August issue of *The Nurses' Journal*. Gifts of books are still being received, and a cordial vote of thanks to those publishers who have generously responded to the appeal made by H.R.H. the President was carried with acclamation at the meeting of the general council on Friday last. It is, however, hoped that large numbers of books will still be contributed, especially when the fact that the library is actually in process of formation is realized by the members of the Association.

### Care of the Sick in Alexandria and Cairo.

### IV.—THE JEWS' HOSPITAL.

This new hospital was the gift of Baron de Menasse, who died before its completion, which was carried out by his son. It contains thirty-five beds, which are none too many, as there are 20,000 Jews in Alexandria. The chief disease from which they suffer appears to be typhus. The nursing is done by two English sisters, who wear red merino dresses with white over-sleeves, aprons, and white lace caps with long strings behind.

The sisters have trained five men to assist in the nursing, and two women, one for day and one for night work. Unfortunately the men cannot be depended upon to give medicine or take temperature without constant supervision, for, as we were told, "they have a provoking way of looking innocently in your face when you convince them of falsehood, and say admiringly, 'How clever of you to find us out.'"

The doctor is an Italian Jew, and he smooths difficulties out of the sisters' way, as he naturally understands his own people.

There are small wards containing three beds, and large ones holding more. The rooms devoted to eye diseases are provided with green nets. All patients are received without payment. The garden is divided, one-half being for men, the other for women. The linen-room looked very neat, the stores being protected by glass doors. The kitchen, too, was very satisfactory, fitted up with an English stove and filters of the best kinds; the man cook has lived in English families, which is good for the nurses, who, of course, do not have the fare of the Jews, *i.e.*, everything cooked in oil, and meat and fowls killed and prepared in a special way. Beef-tea and milk are served in different cups, the first in white and the last in blue. When a patient dies all the water that happens to be in the house is thrown away at once. Three beds are kept at the disposal of the Austrian Consul. The operating-room, though small, is very complete; good table, excellent instruments, all kept in beautiful order. Terraces leading into the garden give the house, from the road, a very pretty appearance, and there is abundance of light and air within.

The sisters admitted that they sometimes found it a little lonely with only Natives and Jews to speak to, but they like their work, and certainly their services are valued.

### The Book World for Women and Nurses.

[We invite Correspondence, Criticism, Enquiries, and Notes on Books likely to interest Women and Nurses. Address, Editor, The Hospital (Nurses' Book World), 428, Strand, W.C.]

### MAGAZINES OF THE MONTH.

The *Pall Mall Magazine* opens its pages this month with the prettily illustrated poem called "A Ballad of an Old Signboard." Another effectively illustrated "Ballad of a Playhouse" is to be found on page 365, which is also attractive in form and substance. "The Romaney's Rest," No. 2 of the series of "Impression" studies, is clever, and suggestively drawn in. "Joan Haste" continues to unfold its history, having now reached its thirty-seventh chapter. Mr. Grant Allan gives us his third paper on "The Evolution of Early Italian Art"; the writer speaks evidently from a full mind, and the subject is both well written and comprehensively treated. But then, most things in the magazine are well written, and we can only repeat that the high standard of the past is being maintained in the present instance in these pages. To meet the increased expenditure necessitated by the purchase of new presses and machinery for the production of the best art work, which the wide circulation of the *Pall Mall Magazine* demands, and to ensure, in the larger editions, the uniform excellence of each individual copy, the price, commencing with the August number, will be 1s. 6d., we are warned in the current volume.

The *Contemporary Review* for July has many excellent articles—"Atavism and Evolution," by Professor Lombroso; and the "High Church Doctrine as to Marriage and Divorce," by Dr. George Serrell; "Ten Years' Postal Progress: An Imperial Plan," by Mr. J. Henniker Heaton. Mr. Charles Roberts' paper on "The Physiology of Recreation" is profitable reading for all those whose consciences are opposed to the physical demands of rest for brain and body. "On Undesirable Information" is an attractively written protest by Mr. E. F. Benson, who boldly denounces the part played by certain modern biographers of public characters. "Why is there this demand for domestic details?" says the author. "Is it part of the pestilence which walks not in darkness but in light, and insists on tearing the veil off everything beautiful? Would a nineteenth century hero find consolation for Leander's death in conducting a post-mortem examination of his body?" Mr. Benson's admonition is a salutary one, and, be it said to their shame, by no means unprovoked on the part of the said biographers.

The *English Illustrated Magazine* has again a lovely calendar of the month. This is not only a pretty, but it is essentially a uniquely useful conception on the part of the editor of the magazine. There are the usual number of short stories, each complete in itself, in these pages, and there are some good papers of a non-fictional order—Mr. Grant Allan's "Moorland Idylls," for instance. "His Summer Stroll" is charmingly written, and the writer's power of observing Nature is further enhanced by his power of expressing such observations. He teaches us to see and to think, not only to casually look at Nature. It would be an instructive lesson to us all to follow the writer in these "Idylls," and be educated in the use of our eyes in everyday matters which surround us in Nature.

## Figures and Tables

**Fig. 37 f1:**
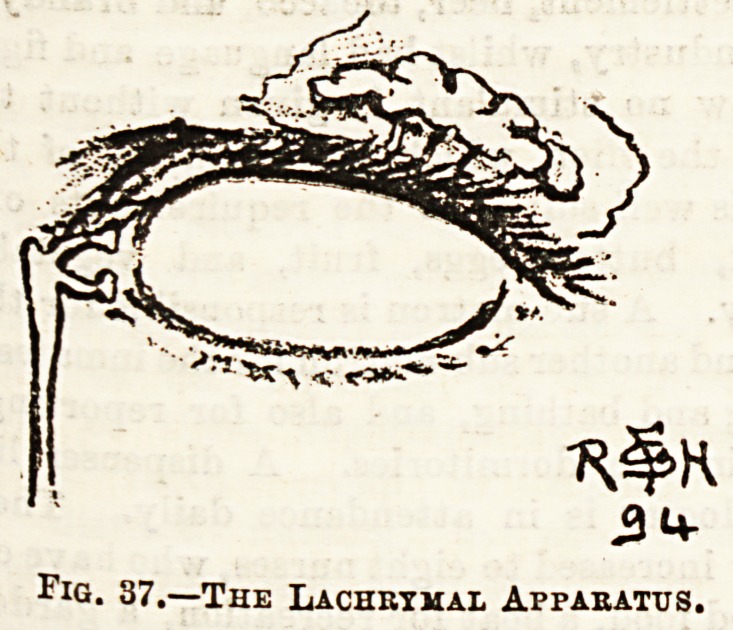


**38 f2:**